# Culturable endophytic fungal communities associated with plants in organic and conventional farming systems and their effects on plant growth

**DOI:** 10.1038/s41598-018-38230-x

**Published:** 2019-02-08

**Authors:** Ye Xia, Mohammad Radhi Sahib, Amna Amna, Stephen Obol Opiyo, Zhenzhen Zhao, Yu Gary Gao

**Affiliations:** 1 Department of Plant Pathology, The Ohio State University, Columbus, OH 43210 USA; 2 Department of Horticulture, University of Kentucky, Lexington, KY 40546 USA; 3 South Centers, The Ohio State University, Piketon, OH 45661 USA; 4 Department of Horticulture and Crop Science, The Ohio State University, Columbus, OH 43210 USA

## Abstract

As compared to organic farming system, conventional farming system relies on higher inputs of synthetic agrochemicals, which may reduce the abundance, diversity, and beneficial effects of plant endophytic fungal communities. This study compares the diversity and abundance of culturable endophytic fungal communities associated with four plant species –corn, tomato, pepper, and watermelon grown in separate organic and conventional fields. In all, 740 fungal isolates were identified, of which 550 were from the organic fields and 190 from the conventional ones. These fungal isolates were grouped into eight orders and 22 species, with the two most abundant species being *Trichoderma sp*. and *Pichia guilliermondi*. The fungal species diversity and abundance were both significantly higher in the organic than in the conventional fields. All the isolated endophytic fungi improved tomato plants’ shoot growth and biomass significantly, as compared with the water control. Six fungal isolates also exhibited activity that enhanced tomato fruit yields. These results suggest that these endophytic fungi might be a considerable boost to sustainable agricultural production, while also reducing the agricultural application of chemicals and thus benefiting the environment and human health.

## Introduction

Plant-associated endophytes, including endophytic fungi, are widely distributed in nature^1^. According to Petrini’s definition, such endophytes include “all organisms inhabiting plant organs that, at some time in their life, can colonize internal plant tissues without causing apparent harm to the host”^2^. Most plants in the world developed associations with microbial endophytes during co-evolution that occurred millions of years ago^3,4^. Endophytic fungi can be beneficial to their host plants through mutualistic symbiosis, but they can also become latent pathogens or saprophytes during certain phases of the hosts’ growth cycles and/or under certain environmental conditions^1^.

During the mutualistic interactions of plants and fungal endophytes, the plants can provide vast and diverse niches for the endophytes^2^. At the same time, many endophytic microbes can help plants with the uptake and utilization of soil nutrients, through their bio-simulation of many compounds and enhancement of their availability. These nutrients have been linked to the promotion of plant growth and development, and thus to the increased plant yields^5,6^. Many endophytic fungi are also capable of improving plants’ resistance to pathogen and insect attacks^7,8^ and their defenses against abiotic stresses, such as those brought on by drought and excessive salt^9^. The interactions between plants and endophytic fungi can also significantly affect the integrity and sustainability of whole agro-ecosystems^3^, and very often, such fungi can produce metabolites and signals involved in these beneficial interactions^5^.

Next-generation sequencing and diverse omics techniques have greatly improved our understanding of microbiome compositions and their functional interactions with host plants. For instance, soil types and plant genotypes have both been shown to play critical roles in the interactions between Arabidopsis plants and their root-associated bacterial endophytes^10–12^. However, the factors affecting such interactions, such as the effects of different agricultural practices on the diversity and functions of plant-associated endophytic fungal communities, require considerable further investigation.

Organic agriculture has rapidly gained in economic importance in the United States since the 1990s, and developed even earlier in Europe^13^. It currently garners considerable attention from farmers, consumers, policymakers, and other stakeholders. The proportion of land dedicated to organic farming has expanded significantly in most regions of the world. Sales of organic products in the U.S. increased fivefold between 1999 and 2013, when they were valued at $72 billion and predicted in the same year to increase further in the future by the Research Institute of Organic Agriculture (FiBL)^14^. The U.S. Department of Agriculture (USDA) defines organic agriculture as “an ecological production management system that promotes and enhances biodiversity, biological cycles, and soil biological activity based on minimal use of off-farm inputs and on management practices that restore, maintain, and enhance ecological harmony”^15^. With regard to labeling, the USDA defines “organic” as denoting products produced under the authority of the Organic Foods Production Act (https://www.nal.usda.gov/afsic/organic-productionorganic-food-information-access-tools). The European Union’s Council Regulation 834/2007 similarly defines organic farming as “an overall system of farm management and food production that combines best environmental practices, a high level of biodiversity, the preservation of natural resources, the application of high animal welfare standards and a production method in line with the preference of certain consumers for products produced using natural substances and processes”^16^. As such, organic farming system usually allows far less application of synthetic chemicals and genetically modified products than traditional agricultural approaches do, as part of an effort to optimize ecological benefits to whole communities, which are conceived of as including not only people, but also the soil, plants, animals, and the natural environment^17,18^. Organic farming also has considerable promise for improving energy efficiency, soil and water conservation, and plants’ tolerance to biotic and abiotic stresse^19^, and for providing the world with higher-quality food despite a changing global climate^20^. In short, organic agricultural system, despite its rapid and recent emergence, has demonstrated its clear potential to benefit the environment and human health^21^.

Since the early 1990s, organic farming has been legally protected in Europe for both consumers and producers by Council Regulation 2092/91. Policymakers and stakeholders have made immense efforts to develop the sustainable organic farming system, which has become very dynamic and complex^18^. The EU’s Common Agricultural Policy (CAP) was designed as a partnership between agriculture and society as well as between Europe and its farmers (https://ec.europa.eu/), and is intended to foster the ongoing development of organic and sustainable agriculture and their principles and values^18,21^. The CAP, like many other agricultural policies and organizations, encourages diverse agricultural practices in the organic farming system, such as the application of companion cropping, green manure, and cover crops. This has significantly improved soil quality and microbial diversity, which in turn have led to higher crop productivity, quality, and prices^18,22^.

In a previous study, we investigated functional associations between culturable bacterial endophytes of plants grown in the organic farming system and conventional farming system^23^. However, systematic studies of the effects of different farming practices on crops’ culturable endophytic fungal communities have been rare. The advantage of the culturable approach is that allows isolation, identification, and further study of the functions and applications of endophytic fungi, which might benefit their host plants’ growth, development, and health^9,24–26^.

Previous study showed that around 69% of crop-yield losses were caused by unfavorable physicochemical environments, such as the inappropriate soil nutrient and water levels^27^.The organic farming practices have been designed to encourage soil and water conservation, reduce chemical pollution, increase the abundance and diversity of soil microbes, and improve the quality and safety of food supplies^19,28^. The present study’s general hypothesis is that the application of chemicals in conventional farming system reduces plant-associated microbiomes’ diversity and abundance, and thus their benefits to plant growth, development, and health.

The two interlinked objectives of this study were therefore 1) to investigate the potentially divergent effects of organic and conventional agricultural practices on culturable plant-associated endophytic fungal communities’ diversity, abundance, and function and 2) to identify culturable fungal isolates that are likely be beneficial to plant growth, health, and yields. As such, its findings could significantly aid the development of novel, efficient, and sustainable strategies for improving crop quality and productivity while reducing the application of harmful chemicals.

## Results

### Community composition and abundance

**Figure 1:**
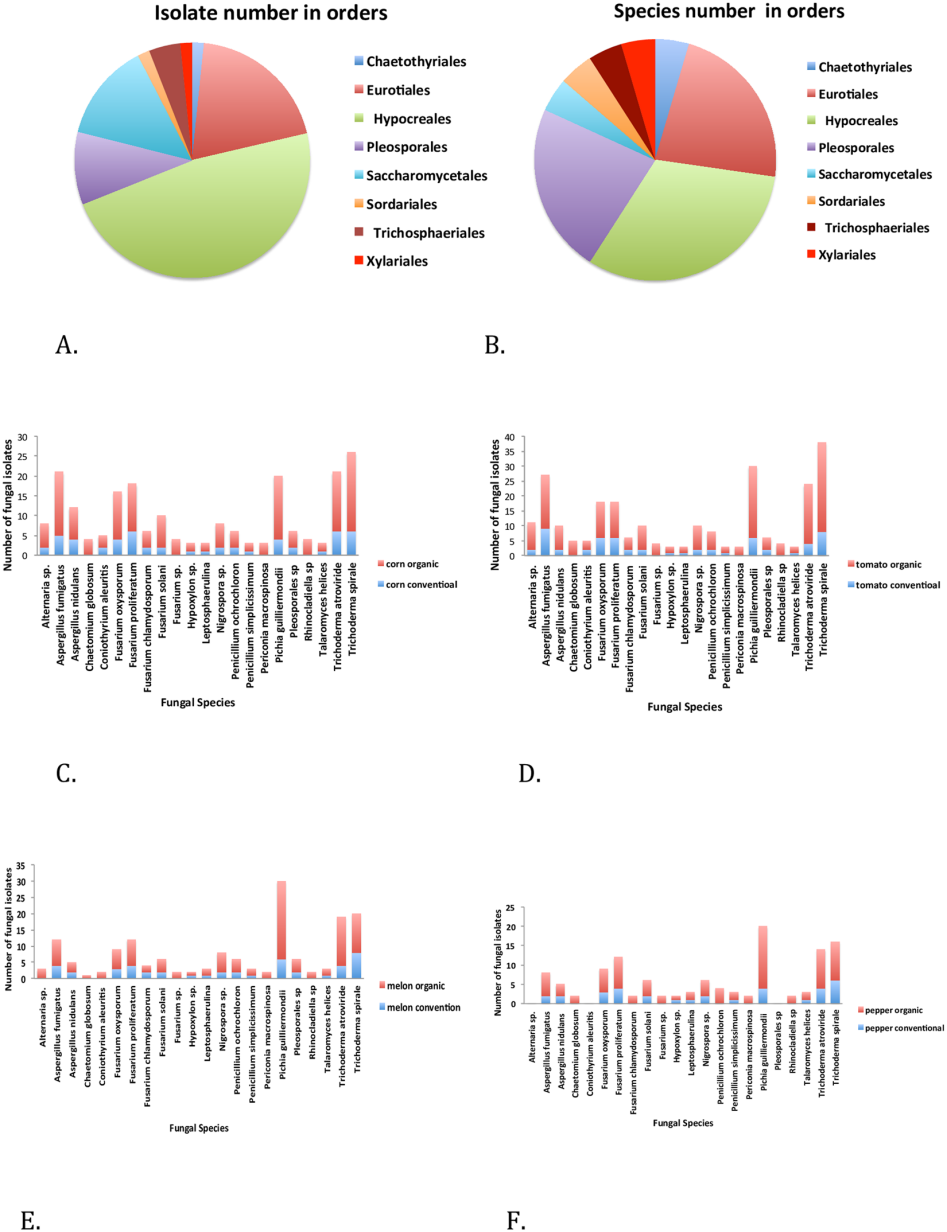
Taxonomic distribution of the endophytic fungi, which were isolated from tomato, corn, watermelon, and pepper plants grown in organic and conventional fields and identified by ITS sequencing. (**A**) Distribution of fungal isolates (n = 740) belonging to each order (n = 8). (**B**) Distribution of fungal species (n = 22) belonging to each order (n = 8). (**C–F)** Fungal species’ distributions separated by different production systems and plant species.

A total of 740 culturable endophytic fungal isolates were obtained from tomato, corn, watermelon, and pepper plants collected from the organic and conventional fields. Of these, 28 different fungal isolates were identified based on the top hits for Internal Transcribed Spacer (ITS) sequences in the National Center for Biotechnology Information (NCBI) database, and morphology phenotypes grown on potato dextrose agar (PDA) plates^4^. The whole samples of 740 fungal isolates were subdivided into 22 different species, which were further classified into eight distinct orders (Fig. 1A,B). The supplementary figure (Fig. S1) shows the phylogenetic tree with the accession numbers of the matched rDNA-ITS sequences of these 28 fungal isolates from the NCBI in parentheses, and the BLASTn expected values and percent-sequence identities in brackets by the method reported previously^29,30^. The results confirm that these isolates are separate species, a conclusion supported by bootstrap values. For example, the *Pleosporales* sp. (GQ923961) and *Pleosporales* sp. (GQ924020) were clustered together with a bootstrap value of 92%. Similarly, *Aspergillus nidulans* (HQ285615) and *Aspergillus nidulans* (EU409807) were in the same group, with a bootstrap value of 97% (Fig. S1).

At the order level, the Hypocreales were the most common, which included 352 isolates and seven species, around 48% and 32% of the total fungal isolates and species, respectively (Fig. 1A,B). The Eurotiales and Saccharomycetales were the second and third most abundant orders, and together constituted approximately 20% and 14% of all isolates and species, respectively (Fig. 1A,B). The other identified orders were the Pleosporales, Trichosphaeriales, Chaetothyriales, Sordariales, and Xylariales (Fig. 1A,B). Interestingly, the most common fungal species across all crops and field types was *Pichia guilliermondi*, followed by *Thrichoderma spirale* and *Thrichoderma atroviride*. However, the number and distribution of fungal species varied considerably by plant species (Fig. 1C–F).

**Figure 2:**
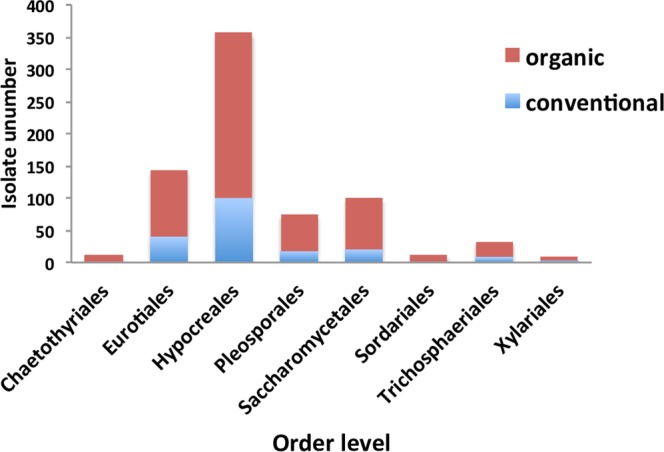
Distribution of the orders of the identified endophytic fungi in organic and conventional farming systems.

The abundance and species diversity of culturable endophytic fungi were both significantly higher in the organic fields than in the conventional ones. Of the 740 total isolates, 550 (74.3% of total) were obtained from plants grown in the organic fields (Fig. 1C–F). A total of 18 fungal species were distributed in both field types, but four species–*Chaetomium globosum*, *Fusarium sp*., *Periconia macrospinosa*, and *Rhinocladiella sp*. were only found in the organic ones (Fig. 1C–F). Similarly, of the eight orders, six were found in both fields, but the Chaetothyriales and Sordariales were unique to the organic fields (Fig. 2). The species diversity of the endophytic fungi associated with corn, pepper, and melon plants in the organic fields was significantly higher than the same plant species in the conventional fields. In the case of tomato plants, such species diversity was also higher in the organic fields, but this difference was not significant (Table 1). The abundance of the endophytic fungal species associated with all four plant species in this study was significantly higher in the organic fields than in the conventional ones (Table 2).

**Table 1:** A comparison of endophytic fungal species diversity (Shannon Index Values) determined in samples obtained from four plant species and multiple types of plant tissues cultivated in conventional vs. organic farming system.

Plant	Factor	Shannon Indices	t-value	P-value
Total	**Con**. **vs**. **Org**	**2**.**55 vs**. **2**.78*	**3**.**27**	<**0**.**001**
Corn	**Con**. **vs**. **Org**	**1**.**84 vs**. **2**.67*	**8**.**32**	<**0**.**001**
Melon	**Con**. **vs**. **Org**	**1**.**93 vs**. **2**.57*	**4**.**54**	<**0**.**001**
Pepper	**Con**. **vs**. **Org**	**1**.**94 vs**. **2**.47*	**4**.**15**	<**0**.**001**
Tomato	Con. vs. Org	2.63 vs. 2.71	0.7	0.48
All	**Shoot vs**. **Root**	**2**.**57 vs**. **2**.77*	**3**.**28**	<**0**.**001**
All	**Shoot vs**. **Seed**	**2**.**57 vs**. **2**.42*	**2**.**28**	<**0**.**05**
All	**Root vs**. **Seed**	**2**.**77 vs**. **2**.42*	**5**.**41**	<**0**.**001**

*Significant differences in Shannon indices of fungal species diversity between the organic and conventional fields and among different plant tissue types (Hutcheson *t-*test, p < 0.05).

**Table 2:** The species abundance of the endophytic fungal isolates from each plant species grown in conventional vs. organic farming system (Hutcheson *t*-test).

Plant Species	Species abundance	Species abundance	P-value
	Conventional	Organic	
Tomato	**0**.**232**	**0**.**768**	<**0**.**001**
Corn	**0**.**239**	**0**.**761**	<**0**.**001**
Melon	**0**.**286**	**0**.**714**	<**0**.**001**
Pepper	**0**.**259**	**0**.**741**	<**0**.**001**

### Fungal species diversity and abundance associated with specific tissues

**Figure 3:**
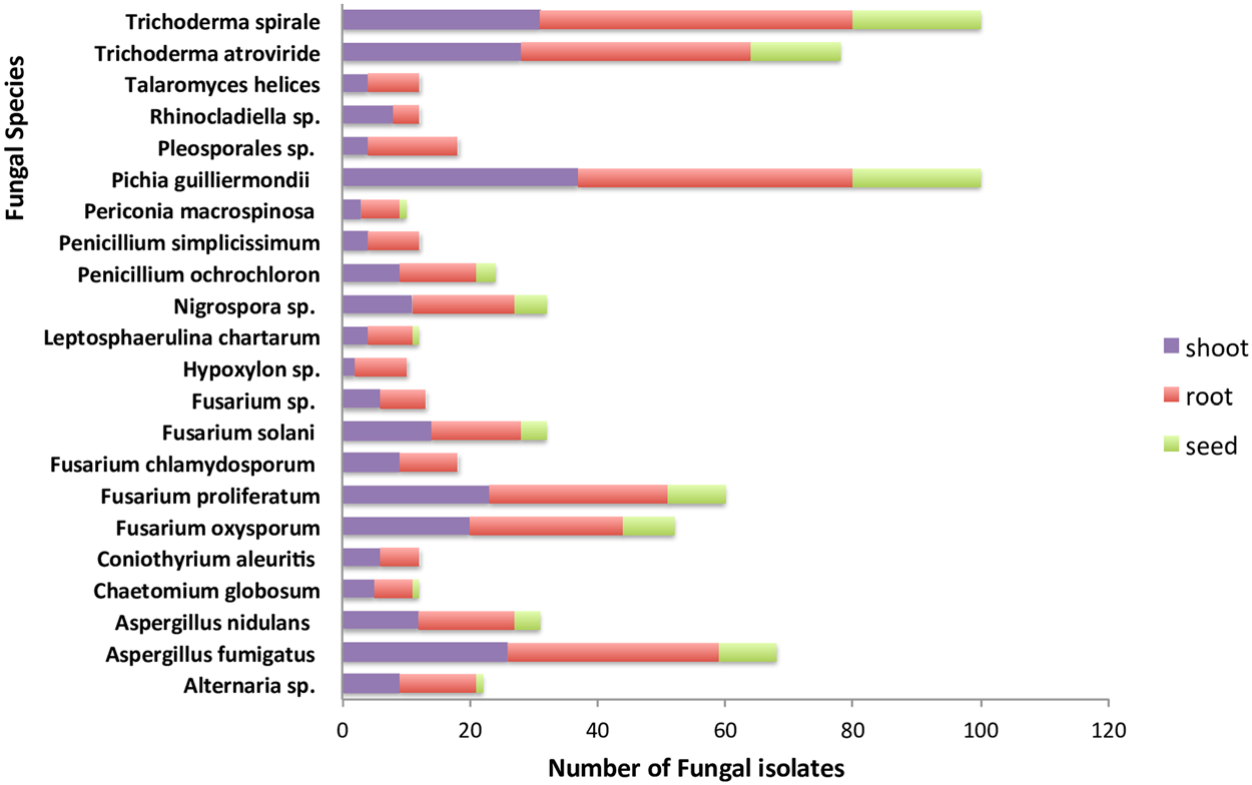
Distribution of the fungal isolates (n = 740) across different plant-tissues as determined by ITS sequencing.

Eight fungal species were only found in plant root and shoot tissues, but not seed tissues, while the other 14 were identified in all three major plant-tissue types (Fig. 3). As measured by the Shannon-Weiner Diversity Index, root tissues in this study displayed the highest level of fungal species diversity and seed tissues had the lowest level (Table 1). More specifically, the root community included 48% of the isolates (n = 365), including all 22 species; the shoot community, 38% of the isolates (n = 275), again including all species; and the seed community, 14% of the isolates (n = 100), which belonged to less than two-thirds of the species (n = 14) (Fig. 3). Analysis of variance (ANOVA) revealed that differences in the abundance of endophytic fungi across shoot, root, and seed tissues in both field types were significant (Table 3).

**Table 3:** The species abundance of endophytic fungal isolates from different plant tissues shows significant differences between convectional and organic production systems, as established through the analysis of variance (ANOVA).

Tissue Type	Species abundance	Species abundance
	Conventional (P-value < 0.05)	Organic (P-value < 0.001)
Shoot	0.55	0.40
Root	0.38	0.46
Seed	0.07	0.14
Total	0.26	0.74

### Fungal isolates showed the promotion activity for plant growth and fruit yields

**Figure 4:**
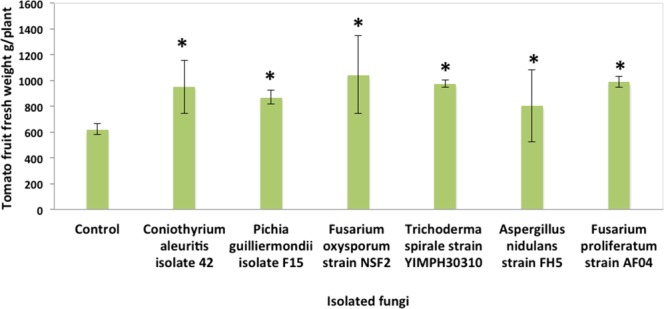
Beneficial effects of six different fungal isolates on tomato fruit fresh weights, as compared with the control condition (water treatment) (*P < 0.05, as established by Dunnett’s test).

As noted above, one of the main aims of this study was to identify endophytic fungi that could be cultured and applied to plants to enhance their growth, health, and yields. Therefore, we tested 28 different fungal isolates for their ability to enhance tomato plants’ shoot growth and biomass. All 28 isolates were found to do so: with the plants’ heights, fresh weights, and dry weights all significantly greater than those of the plants treated with water control (Tables S1–S3). Detailed information about the ITS sequences and NCBI accession numbers of these fungi is provided in Figure S1 and Table S4. Among the top 10 fungi in terms of their promotion of tomato shoot height, six also were found to improve tomato fruit yield. These were *Coniothyrium aleuritis* isolate 42, *Pichia guilliermondii* isolate F15, *Fusarium oxysporum* strain NSF2, *Fusarium proliferatum* strain AF04, *Aspergillus nidulans* strain FH5, and *Trichoderma spirale* strain YIMPH30310 (Fig. 4).

## Discussion

Organic farming practices have developed rapidly worldwide over the past 20 years because of their lower inputs of chemicals and their benefits to crop health and the environment^14,31^. In particular, these practices directly affect soil microbial abundance, diversity, and functions, and thus are likely to be associated with the increased soil health, enhanced plant growth and yields, and improved plant resistance to biotic and abiotic stresses^13^.

The present study revealed that the abundance of endophytic fungal species in the sampled organic fields was significantly higher than that in the sampled conventional ones for all four studied plant species. It was also observed that the plant-associated endophytic fungal community had significantly higher species diversity in the organic fields. The data also suggested that these two agricultural systems differentially influenced the abundance and diversity of the plant-associated endophytic fungal community, and potentially its impacts on plant growth and yields. This echoes previous studies’ findings that fungal endophytes of grapevines and soybeans had higher abundance and species diversity in organic fields than in the conventional fields^32,33^.

In addition, the present study’s results demonstrate that endophytic fungal species’ diversity and abundance differed across different plant tissues, being significantly higher in roots than in shoots or seeds. This might be because roots are the interfaces that connect plants with the soil and the soil-associated microbiome, including some soil microbes that could potentially be plant endophytes^34,35^. Similar results have been reported previously: for example, in regard to the herbaceous grassland plants, medicinal plant *Kadsura angustifolia*, and rice plants^36–38^. Prior studies have also shown, however, that fungal endophytes can be more abundant and diverse in plant tissues other than roots^39,40^. The reason for this discrepancy might be that the distribution of fungal endophytes across different tissue types is affected by different plant species, growth stages, soil types, and environmental conditions^41^.

One of our main aims for this study was to identify the beneficial plant-associated endophytic fungi to increase plant growth, heath, and yield by a sustainable approach. The two most abundant fungal species isolated in this study were *Pichia guillermondii* and *Trichoderma sp*. in total, which were isolated from both the organic and conventional fields in our study. The beneficial effects of *Pichia guillermondii* and *Trichoderma sp*. on enhancing plants’ growth, yields, and resistance to biotic stress have been extensively reported by previous researchers^24,25,42,43^. For example, several *P*. *guillermondii* strains had been reported to significantly inhibit the growth of *Penicillium digitatum*, the pathogen that causes green-mold diseases in citrus and many other crops. Moreover, after treatment with these *P*. *guilliermondii* strains, the peroxidase activity in the flavedo tissues of citrus was significantly higher than that in the untreated controls, and the related activity was associated with the increased resistance to *Penicillium digitatum*. Thus, *P*. *guilliermondii* could potentially trigger plants’ defense mechanisms against pathogen attacks^42^. Additionally, *P*. *guilliermondii* have been shown to promote the growth of diverse plants, such as chili peppers^24^. *Trichoderma sp*., meanwhile, have been widely found to promote plant growth and inhibit a range of plant pathogens, including *Sclerotium rolfsii*, *Verticillium dahlia*, and *Fusarium sp.*^25,43–45^. In addition to promoting tomato plants’ growth, *T*. *atroviride* and *T*. *harzianum* have been reported to activate the plants’ defense-related hormones, such as the salicylic acid (SA) pathway even without the presence of pathogens. They could also activate the jasmonic acid (JA) signaling pathway after attack by the fungus *Botrytis cinera*^43^. *T*. *spirlae* fungi have been identified as phosphate solubilizers and could promote the growth of plants, including eggplants under field conditions^25^.

The six fungal isolates in current study associated with the highest increases in tomato growth and yields belonged to very diverse orders, i.e., the Eurotiales, Pleosporales, Hypocreales, and Saccharomycetales. This implies that a very diverse range of endophytic fungi have considerable potential to improve plant yields and health under natural conditions. Beneficial fungi may increase plants’ growth and yield via various mechanisms: for example, through increasing their uptake and utilization of nutrients, such as nitrogen, phosphate, and iron; promoting their growth hormone production; and activating the genes involved in their growth and development^5,24^. However, the precise mechanisms whereby these six endophytic fungal isolates can improve tomato plants’ growth and yields merit further investigation. It is expected that the outcomes of such future research will lead to the development of a more efficient strategy for utilizing fungal endophytes in sustainable agriculture.

As noted above, plants’ endophytic fungal communities can change in response to diverse factors, including their growth stage and environmental conditions^46^. The present findings highlight that organic as opposed to conventional practices could increase the abundance and species diversity of culturable plant-associated endophytic fungi. Further study, using next-generation sequencing approaches, of the diversity and abundance of the endophytic fungal microbiome in plants grown in organic versus conventional systems, is also likely to improve our understanding of the obligate and unculturable endophytic fungi in the community. It would also be worthwhile to enhance microbial interactions at the community level by creating synthetic beneficial microbial communities from isolated culturable fungal microbes, as this might benefit plants more efficiently than single microbe would.

More comprehensive studies of the effects of various organic-farming practices, such as the use of cover crops, rotation, tillage, or crop integration, could help identify the best practices for promoting the abundance and diversity of the soil- and plant-associated microbiomes, which in turn should benefit plant growth and yields. Such further investigations would also facilitate more efficient applications of beneficial endophytic microbes in organic and sustainable agriculture practices.

Prior findings discovered that the differences in agricultural practices could strongly influence the abundance and composition of soil-associated microbiomes^47–50^. One reason for this is that the differences in water use across different farming types can affect soil environment in complex and interactive ways. For instance, the survival and growth of microbes in soil can be directly affected by the soil water content, which can also influence microbes’ access to nutrients that are essential for their survival^47–49^. Another key factor is fertilizer application. For example, the amount and quality of organic fertilizers could both play critical roles in soil microbial diversity^50^. Microbial-community differences between organic and conventional farming systems could also be more significant when pesticide application is large, soil-tillage operations occur, and cropping systems lack soil-replenishing crops, such as legumes^50^. Since some soil microbes might potentially become plant endophytes^34,35^, these agricultural practices could also affect the plant-associated endophytic microbial community, as our present study.

It is important to remember, however, that the soil- and plant-associated microbiome is not limited to fungi, but also includes bacteria and other microorganisms, which might not be culturable in certain growth media. Yet, these microbes’ interactions with one another may affect not only their own survival, but the overall community’s impact on plant and soil health, in ways that remain under-researched and poorly understood^34,51^.

Some prior research has suggested that tillage practices could affect the C:N ratio of the microbial biomass in soil, but only cause significant differences in fungal dominance over bacterial dominance when soil aggregations change^52,53^. One study has also shown that some plant endophytic fungi have a greater capability to solubilize inorganic phosphates, and produce more active enzymes, than endophytic bacteria do, meaning that the former could lead to higher plant biomass than the latter^54^. In addition, fungi have exhibited desiccation responses in soil, which might indicate their higher degree of resistance to water-availability changes, as compared to bacteria^55^. However, further study should explore the effects and functional mechanisms of fungi versus bacteria in different crops and agricultural systems under a variety of environmental conditions.

Taken as a whole, the present study’s results indicate that organic farming practices can have significant positive effects on the abundance and diversity of plant-associated culturable endophytic fungal communities. All the endophytic fungi that were isolated and cultured during this study exhibited significant beneficial effects on tomato plants’ shoot growth and biomass, and six were also found to significantly increase the plants’ fruit yield following soil-drench treatment with the broth of fungal spores and mycelia. The results suggest the strong potential of these endophytic fungal microbes to benefit agriculture, not only through the promotion of crop growth and yields, but also through the reduction of chemical inputs. However, further investigation of the mechanisms by which the beneficial functions of these fungal endophytes occur, and how to utilize them efficiently in practice, will be critical to the success of their further application in sustainable agricultural production.

## Methods

### Plant sample collections

The corn (*Zea mays var*. *Silver Queen*), tomato (*Solanum lycopersicum var*. *Big Beef*), watermelon (*Citrullus lanatus var*. *Sugar Baby*), and pepper (*Piper nigrum var*. *Aristotle*) plants were grown in the organic and conventional fields separately in the Horticulture Research Farm of University of Kentucky (Lexington, USA, lat.38◦ 3′N, long.84◦ 30′W). Eighty plants of corn, tomato, watermelon, and pepper (20 plants for each plant species) were collected separately from the organic and conventional fields during the summer season in 2014. The related research farm has been in operation for more than 25 years with the conventional practice and the organic practice field was developed from the same farm, which had been established and certified for more than 10 years according to the National Organic Program’s Organic Standards^15^.

### Isolation of the endophytic fungi from the plant shoot, root, and seed tissues

The approach to isolate the endophtyic fungi from the plants was followed by the widely reported method through the plant surface sterilization^23,56–58^. The same weight of 0.5 gram of shoots (leaf/stem) and root segments with approximately 1 cm–1.5 cm in length were taken from the collected 20 plants for four different plant species in both the organic and conventional fields. These segments were washed with deionized water (dH2O) to remove the remnant soil and debris. The segments were immersed into 20% Clorox bleach (sodium hypochlorite) containing 0.1% Tween 20 (Sigma-Aldrich Co., St. Louis, Mo.) in sterile dH_2_O for 15 mins, rinsed with 95% ethanol (EtOH) for 2 mins, and then serially rinsed 5 times in sterile dH_2_O^9,56–58^. The same amounts of seeds were excised from the lemma and palea of the collected plants, cut into two parts, and then surface sterilized as above. To test the efficacy of this method, the final step dH_2_O rinsed off the plants was randomly collected and plated on the PDA (Potato Dextrose Agar) plates and incubated at 26 °C for 15 days to confirm the absence of any microbial growth after the disinfection.

All the cut segments of different tissues from each plant were placed separately on the plates with the PDA medium containing the ampicillin, penicillin, and streptomycin (30 μg/ml) to inhibit the bacterial growth (Fisher Scientific, USA). The plates were then incubated in the growth chamber with the setting temperature at 26 °C for 7–12 days and the occurrence number of individual fugal colonies emerged were recorded separately from different tissues and different plants to further calculate the fungal abundance and species diversity^23,56,59^. The single colonies were cultured separately on plates with the repeats for 2–3 times to get the pure culture for further study.

### DNA extractions, ITS rDNA gene amplification, sequencing, and OTUs identification

The DNA from the purified fungal isolates grown in PDA plates was extracted by using the Zymo Research fungal/bacterial DNA mini-prep kit (Zymo Research, CA, USA) following the manufacturer’s instruction. The ITS (Internal Transcribed Spacer) region is the most widely sequenced DNA region for the fungi and it has higher degree of variation than most of the other genic regions of rDNA^60,61^. Therefore, the ITS sequencing method had been selected for our study. The rDNA amplification of the ITS region was performed in a 50 µl reaction mixture, which included 3 µl DNA template (1–20 ng), 50 µM of primers ITS1 (5′- TCCGTAGGTGAACCTGCGG -3′) and ITS4 (5′- TCCTCCGCTTATTGATATGC -3′)^29,34,60–64^, 3 mM Mgcl_2,_ 3 mM dNTPs, 5 µl of *Taq* buffer, and 1 U *Taq* DNA polymerase (Fermentas Inc., MD, USA). The PCR amplification was performed on a cycler PCR machine (Bio-Rad Labortories, CA, USA) with the initial denaturation at 94 °C for 5 mins, followed by 30 cycles of amplification (94 °C for 1 min, 55 °C for 1 min, 72 °C for 1 min) and an extension step (72 °C for 5 mins)^23^. The PCR products around 600 to 700 bps were purified using the Zymo PCR purification kit (Zymo Research, USA) and quantified by a nano-drop spectrophotometer. The purified products had been sent to the eRAMP genome center at The Ohio State University for sequencing by the Sanger sequencing method^65^. The sequences had been blasted through the BLASTn in the NCBI (The National Center for Biotechnology Information, https://blast.ncbi.nlm.nih.gov/Blast.cgi) Databases and the top hits were used to identify the most suitable OTUs (Operational Taxonomic Units).

### Data organization and statistical analysis

As well as being sorted by species, the fungal isolates were grouped into a higher taxonomic level (orders); and their relative abundance was evaluated according across three major types of plant tissues and two general types of agricultural systems (conventional vs. organic). Multiple alignments of the rDNA-ITS sequences were generated using multiple-alignment fast Fourier transform^66^ and the related phylogenetic tree was constructed by the maximum-likelihood method RAxM^67^. Species diversity was calculated using Shannon-Weiner Diversity Index values^68,69^ and the Hutcheson t-test and ANOVA were used to compare the relative abundance of fungal species across shoot, root, and seed tissues and between the organic and conventional systems^69,70^. Dunnett’s test was used to analyze the effects of different fungal isolates on tomato plants’ shoot growth, weights, and fruit yields^71^.

### Test of the plant growth promotion and yield enhancement activities of different endophytic fungal isolates

The tomato seeds (var. OH981205) were washed with 95% EtOH in dH_2_O for 2 mins and were soaked with 30% bleach (NaOCl) and 5% sodium dodecyl sulfate (SDS) in dH_2_O for 20 mins. The seeds were further rinsed with the sterilized dH_2_O 5 times and incubated at 4 °C in a cold room for 24 hrs. The washed tomato seeds were then sown into pots that contained the Pro-Mix potting media (Premier Horticulture Inc., PA, USA). The pots were kept in a greenhouse with the constant temperature of 24 °C with 16 hrs of light followed by 8 hrs of darkness for 7 days for the seed germination^23^. Then, the tomato seeds were inoculated with the fungal inoculation mixture containing fugal mycelia and spores (if present) in 100 ml water in each pot as described below and previously^62^. Each pot contained one tomato seed and six pots were used for each fugal treatment and water treatment as controls. The related experiments had been repeated for three times.

For the fungal inoculation mixture preparation: the pure and different individual 28 fungal isolates were maintained and cultured on the petri dishes filled with PDA media. For each endophytic fungal isolate, three fugal plugs with diameter around 0.6 cm were taken from the culturing PDA plates and put in the center of three new PDA medium plates separately and grown in a 26 °C incubator for 7 days. Then the fungal spores (if present) and mycelia from three plates were washed off with the sterilized water and put into the sterilized flasks and diluted to a total of 600 ml. About 100 ml of the fungal broth containing the fungal spores (if present) and mycelia were applied to the top of the potting mix in each pot containing the tomato seed underneath; and 600 ml water treatment were used as controls in separate 6 pots with 100 ml for each pot^62^.

All the pots were kept in a greenhouse with a constant temperature of 24 °C and exposed to 16 hrs of light per 24-h period. The tomato plants’ shoot growth (height) and biomass (fresh weight, and dry weight after being placed in an oven at 70 °C for 48 hrs) were measured at around 10-week growth stage. The experiments were repeated three times, and the data from 18 plants in each treatment condition were pooled together for further analysis. The comparison of tomato plants’ above-ground shoot heights, dry weights, and fresh weights across each fungal treatment and water-treatment control were analyzed by using Dunnett’s test^71^.

The top 10 fungal isolates in terms of their positive impact on tomato plants’ shoot heights were selected for further testing of their effects on tomato fruit yields. Six plants in each of the 11 treatment conditions (i.e., for each of the top 10 fungi plus the water control) were chosen for measuring. The combined weight of several batches of tomatoes harvested from each plant during the entire growing season was counted as the total tomato fruit yields. The experiments were repeated three times and the data for 18 plants were used for further analysis.

## Supplementary information

 Supplementary information accompanies this paper at https://doi.org/10.1038/s41598-018-38230-x. 

## Data Availability

The authors claim that all data generated or analyzed during this study are included in this article and its supplementary information files.
