# Supplementary material for: Culturable endophytic fungal communities associated with plants in organic and conventional farming systems and their effects on plant growth
Source: Sci Rep. 2019 Feb 8;9:1669. doi: 10.1038/s41598-018-38230-x (PMC6368545; doi:10.1038/s41598-018-38230-x)
Supplement: Supplementary file 1 — Supplementary information accompanies this paper at https://doi.org/10.1038/s41598-018-38230-x. [file 41598_2018_38230_MOESM1_ESM.docx]

**Culturable endophytic fungal communities associated with plants in organic and conventional farming systems and their effects on plant growth**

^1^* Ye Xia, ^2^Mohammad Radhi Sahib, ^1^Amna Amna, ^1^Stephen Obol Opiyo, ^1^Zhenzhen Zhao, ^3,4^ Yu Gary Gao

**
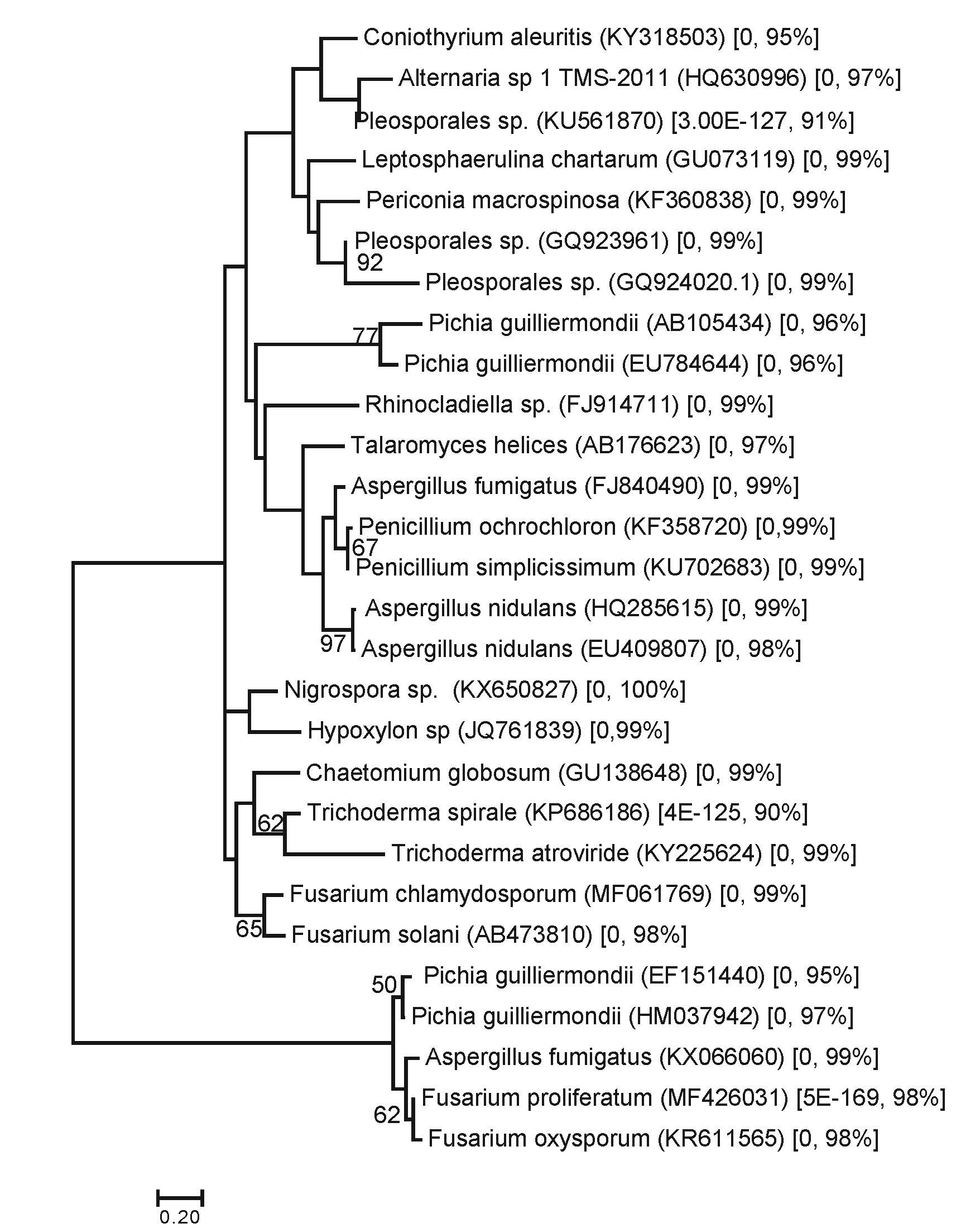
**

**Figure S1. Maximum-likelihood phylogenetic tree of ITS sequences.**

The accession numbers of the NCBI sequences that matched the ITS sequences are shown in parentheses. The expect values (E-values) and percent sequence identity between the ITS sequences and the matched sequences in NCBI appear in brackets. Only bootstrap values $\geq$50 are shown on the phylogenetic tree.

**Table S1. Increases in tomato-shoot heights associated with all isolated endophytic fungi, as compared with the control condition (water treatment).**

**(* P<0.05, as established by Dunnett’s test).**

**Table S2. Increases in tomato shoots’ fresh weights, excluding fruits, associated with all isolated endophytic fungi, as compared with the control condition (water treatment) (* P<0.05, as established by Dunnett’s test).**

|  | Isolated Fungi NCBI  Accession number | Fresh weight (g/plant) | SD | P-value |
| --- | --- | --- | --- | --- |
| 1 | **EF151440.1 *Pichia guilliermondii* isolate F15** | **117.71*** | **7.50** | **<0.05** |
| 2 | **KY318503.1 *Coniothyrium aleuritis* isolate 42** | **123.77*** | **10.96** | **<0.05** |
| 3 | **HQ285615.1  *Aspergillus nidulans* strain KCCM60326** | **125.50*** | **10.93** | **<0.05** |
| 4 | **HQ630996.1  *Alternaria sp. 1 TMS*-2011 voucher MS2-18** | **122.20*** | **10.35** | **<0.05** |
| 5 | **KR611565.1 *Fusarium oxysporum* strain NSF2** | **108.95*** | **11.19** | **<0.05** |
| 6 | **MF426031.1 *Fusarium proliferatum* strain AF04** | **110.01*** | **10.11** | **<0.05** |
| 7 | **KP686186.1 *Trichoderma spirale* strain YIMPH30310** | **104.64*** | **11.31** | **<0.05** |
| 8 | **EU409807.1  *Aspergillus nidulans* strain FH5** | **112.15*** | **11.40** | **<0.05** |
| 9 | **KF358720.1 *Penicillium ochrochloron* strain PFR8** | **108.10*** | **12.24** | **<0.05** |
| 10 | **AB105434.1 *Pichia guilliermondii* strain IAM 14500** | **107.38*** | **9.19** | **<0.05** |
| 11 | **KX650827.1** ***Nigrospora sp. isolate DL2*** | **108.95*** | **9.00** | **<0.05** |
| 12 | **KF360838.1 *Periconia macrospinosa* strain KS00113** | **106.45*** | **8.34** | **<0.05** |
| 13 | **KX066060.1 *Aspergillus fumigatus* strain FZ 18** | **107.10*** | **5.95** | **<0.05** |
| 14 | **KY225624.1 *Trichoderma atroviride* strain QT21978** | **106.55*** | **4.53** | **<0.05** |
| 15 | **GQ924020.1 *Pleosporales* clone K1Fc722H** | **98.97*** | **7.58** | **<0.05** |
| 16 | **FJ914711.1 *Rhinocladiella* sp. HSAUP074099** | **97.23*** | **9.88** | **<0.05** |
| 17 | **AB176623.1 *Talaromyces helices* strain CBS 652.66** | **92.04*** | **11.80** | **<0.05** |
| 18 | **KU561870 *Pleosporales sp. isolate EDSHB10*** | **94.27*** | **10.17** | **<0.05** |
| 19 | **JQ761839.1 *Hypoxylon* sp. genotype 520 isolate NC1198** | **92.47*** | **8.84** | **<0.05** |
| 20 | **GQ923961.1 *Pleosporales* sp. G9i87H** | **96.26*** | **7.64** | **<0.05** |
| 21 | **EU784644.1 *Pichia guilliermondii* strain CXF-1** | **100.33*** | **10.23** | **<0.05** |
| 22 | **MF061769.1 *Fusarium chlamydosporum* strain SCAU107** | **99.62*** | **8.39** | **<0.05** |
| 23 | **HM037942.1 *Pichia guilliermondii* strain wxm69** | **103.95*** | **10.43** | **<0.05** |
| 24 | **FJ840490.1 *Aspergillus fumigatus* strain FS160** | **103.37*** | **10.43** | **<0.05** |
| 25 | **KU702683.1 *Penicillium simplicissimum* isolate BK227** | **103.08*** | **9.24** | **<0.05** |
| 26 | **GU073119.1 *Leptosphaerulina chartarum* strain DH08111** | **104.12*** | **10.95** | **<0.05** |
| 27 | **AB473810.1 *Fusarium solani strain THIF01*** | **103.52*** | **8.37** | **<0.05** |
| 28 | **GU138648.1 *Chaetomium globosum* strain PF-1** | **102.44*** | **7.54** | **<0.05** |
| 29 | **Mock** | **77.69** | **7.20** |  |

**Table S3. Increases in tomato shoots’ dry weights, excluding fruits, associated with all isolated endophytic fungi, as compared with the control condition (water treatment) (* P<0.05, as established by Dunnett’s test).**

|  | Isolated Fungi NCBI  Accession number | Dry weight (g/plant) | SD | P-value |
| --- | --- | --- | --- | --- |
| 1 | **EF151440.1 *Pichia guilliermondii* isolate F15** | **17.6*** | **1.58** | **<0.05** |
| 2 | **KY318503.1 *Coniothyrium aleuritis* isolate 42** | **18.13*** | **1.29** | **<0.05** |
| 3 | **HQ285615.1  *Aspergillus nidulans* strain KCCM60326** | **17.70*** | **1.45** | **<0.05** |
| 4 | **HQ630996.1  *Alternaria sp*. 1 TMS-2011 voucher MS2-18** | **19.02*** | **1.91** | **<0.05** |
| 5 | **KR611565.1 *Fusarium oxysporum* strain NSF2** | **17.13*** | **1.60** | **<0.05** |
| 6 | **MF426031.1 *Fusarium proliferatum* strain AF04** | **16.77*** | **1.63** | **<0.05** |
| 7 | **KP686186.1 *Trichoderma spirale* strain YIMPH30310** | **17.24** | **1.53** | **<0.05** |
| 8 | **EU409807.1  *Aspergillus nidulans* strain FH5** | **16.75*** | **1.33** | **<0.05** |
| 9 | **KF KF358720.1 *Penicillium ochrochloron* strain PFR8** | **16.49*** | **1.17** | **<0.05** |
| 10 | **AB105434.1 *Pichia guilliermondii* strain IAM 14500** | **15.88*** | **0.88** | **<0.05** |
| 11 | **KX650827.1** ***Nigrospora sp. isolate DL2*** | **15.98** | **0.84** | **<0.05** |
| 12 | **KF360838.1 *Periconia macrospinosa* strain KS00113** | **17.27** | **1.07** | **<0.05** |
| 13 | **KX066060.1 *Aspergillus fumigatus* strain FZ 18** | **16.61** | **0.53** | **<0.05** |
| 14 | **KY225624.1 *Trichoderma atroviride* strain QT21978** | **14.71*** | **0.95** | **<0.05** |
| 15 | **GQ924020.1 *Pleosporales* clone K1Fc722H** | **13.92*** | **0.73** | **<0.05** |
| 16 | **FJ914711.1  *Rhinocladiella* sp. HSAUP074099** | **14.14*** | **0.62** | **<0.05** |
| 17 | **AB176623.1 *Talaromyces helices* strain CBS 652.66** | **13.91*** | **0.81** | **<0.05** |
| 18 | **KU561870 *Pleosporales sp. isolate EDSHB10*** | **14.03*** | **0.72** | **<0.05** |
| 19 | **JQ761839.1 *Hypoxylon* sp. genotype 520 isolate NC1198** | **13.89*** | **0.66** | **<0.05** |
| 20 | **GQ923961.1 *Pleosporales* sp. G9i87H** | **13.79*** | **0.70** | **<0.05** |
| 21 | **EU784644.1 *Pichia guilliermondii* strain CXF-1** | **14.11*** | **0.59** | **<0.05** |
| 22 | **MF061769.1 *Fusarium chlamydosporum* strain SCAU107** | **13.77*** | **0.75** | **<0.05** |
| 23 | **HM037942.1 *Pichia guilliermondii* strain wxm69** | **15.07*** | **0.71** | **<0.05** |
| 24 | **FJ840490.1 *Aspergillus fumigatus* strain FS160** | **14.77*** | **2.36** | **<0.05** |
| 25 | **KU702683.1 *Penicillium simplicissimum* isolate BK227** | **13.62*** | **0.72** | **<0.05** |
| 26 | **GU073119.1 *Leptosphaerulina chartarum* strain DH08111** | **14.28*** | **0.86** | **<0.05** |
| 27 | **AB473810.1 *Fusarium solani strain THIF01*** | **13.42*** | **1.17** | **<0.05** |
| 28 | **GU138648.1 *Chaetomium globosum* strain PF-1** | **13.77*** | **0.85** | **<0.05** |
| 29 | **Mock** | **10.90** | **1.45** |  |

**Table S4. ITS sequences and NCBI accession numbers of the endophytic fungi identified in this study, as listed in Tables S1-S3.**

**1. EF151440.1 *Pichia guilliermondii* isolate F15**

GCGCTTACTGCGCGGCGAAAACCTTACACACAGTGTCTTTTTGATACAGAACTCTTGCTTTGGTTTGGCCTAGAGATAGGTTGGGCCAGAGGTTTAACAAAACACAATTTAATTATTTTTACAGTTAGTCAAATTTTGAATTAATCTTCAAAACTTTCAACAACGGATCTCTTGGTTCTCGCATCGATGAAGAACGCAGCGAAATGCGATAAGTAATATGAATTGCAGATTTTCGTGAATCATCGAATCTTTGAACGCACATTGCGCCCTCTGGTATTCCAGAGGGCATGCCTGTTTGAGCGTCATTTCTCTCTCAAACCCCCGGGTTTGGTATTGAGTGATACTCTTAGTCGGACTAGGCGTTTGCTTGAAAAGTATTGGCATGGGTAGTACTAGATAGTGCTGTCGACCTCTCAATGTATTAGGTTTATCCAACTCGTTGAATGGTGTGGCGGGATATTTCTGGTATTGTTGGCCCGGCCTTACAACAACCAAACAAGTTTGACCTCAAATCAGGTAGGAATACCCGCTGAACTTAAGCATATCAATACCGGAGGAAATAAANCNTTACNGTATTCTTTTGCCAGCGCTTAACTGCGCGGGAAAAAAGCTTTAATTCGGCGTCTTTTTGATACAAAATTCTTGCTTTAGTTTAGGCCTAGAGATAGGCTGGGGCCGGAAGTTTAACAAAAACAAATTTAATTATTTTTACATTTAGGCAAATTTGCAAATTAATCNTCAAAAATTGCGACGAACTCNTGGGTTCTTCCTCCGCGACCCNGGGACGCCGTAAGTAATATGAAATTGCAGATTTTCGGGATCTC

**2.KY318503.1 *Coniothyrium aleuritis* isolate 42**

GGCTTTGCCTGCTATCTCTTACCCATGTCTTTTGAGTACCTTCGTTTCCTCGGCGGGTCCGCCCGCCGATTGGACACATTTAAACCCTTTGTAGTTGCAATCAGCGTCTGAAAAACTTTAATAGTTACAACTTTCAACAACGGATCTCTTGGTTCTGGCATCGATGAAGAACGCAGCGAAATGCGATAAGTAGTGTGAATTGCAGAATTCAGTGAATCATCGAATCTTTGAACGCACATTGCGCCCCTTGGTATTCCATGGGGCATGCCTGTTCGAGCGTCATTTGTACCTTCAAGCTTTGCTTGGTGTTGGGTGTTTGTCTCGCCTCTGCGCGCAGACTCGCCTCAAAACAATTGGCAGCCGGCGTATTGATTTCGGAGCGCAGTACATCTCGCGCTTTGCACTCATAACGACGACGTCCAAAAAGTACATTTTTTACACTCTTGACCTCGGATCAGGTAGGGATACCCGCTGAACTTAAGCATATCAATAAGCGGNNNNGAATCTTACCAANAGTTGTAGGCTTTGCCTGCTATCTCTTACCCCTGTCTTTTGAGTACCATCGCTTCCTCGGCGGGACCGCCCGACGATTGGACCGGCATAAAACCT

**3. HQ285615.1 *Aspergillus nidulans* strain KCCM60326**

CGCNNACCTCCCACCCGTGANTACNTAACACTGTTGCTTCGGCGGGGAGCCCCCCAGGGGCGAGCCGCCGGGGACCACTGAACTTCATGCCTGAGAGTGATGCAGTCTGAGCCTGAATACAAATCAGTCAAAACTTTCAACAATGGATCTCTTGGTTCCGGCATCGATGAAGAACGCAGCGAACTGCGATAAGTAATGTGAATTGCAGAATTCAGTGAATCATCGAGTCTTTGAACGCACATTGCGCCCCCTGGCATTCCGGGGGGCATGCCTGTCCGAGCGTCATTGCTGCCCTCAAGCCCGGCTTGTGTGTTGGGTCGTCGTCCCCCCCGGGGGACGGGCCCGAAAGGCAGCGGCGGCACCGTGTCCGGTCCTCGAGCGTATGGGGCTTTGTCACCCGCTCGATTAGGGCCGGCCGGGCGCCAGCCGGCGTCTCCAACCTTATTTTTCTCAGGTTGACCTCNGATCAGGTAGGGAAACCCGCTGAACTTTACCATATCANTAAGGGGAAGAANNCNTTAANGGCTCGCAGGCGATTTCGTAANNCTGATGTGAAANNCCCCCCGTAAACCGGNNATGTTCATTGGAAACTAGGAA

**4. HQ630996.1 *Alternaria sp. 1* TMS-2011 voucher MS2-18**

CTTTATCTATTTGGAGTGTACCTGCGTGCGAAGCCATAACTCTGTGGTGAAAGACGCCGATTGGACGTGCCTNTNCTTTTTTGTAATTGGATCAGGCGTCTGAAAATAATCTAATTATTTACAACTTTCAACAACGGATCTCTTGGTTCTGGCATCGATGAAGAACGCAGCGAAATGCGATAAGTAGTGTGAATTGCAGAATTCAGTGAATCATCGAATCTTTGAACGCACATTGCGCCCCTTGGTATTCCATGGGGCATGCCTGTTCGAGCGTCATTTGTACCCTCAAGCTTTGCTTGGTGTTGGGCGTCTTGTCGTATTACGACTCGCCTTAAATACATTGGCAGCCGGCACTTTGGCCTAGGAGCGCAGCACATTTTGCGATCGTAGCCCGTTGTACTGGCGTCCATCAAGAACATTTACCACGTTTGACCTCGGATCAGGTAGGGATACCCGCTGAACTTAAGCATATCAATAAGCGGAGGGATGTCACTGATCGCACTTAACTGTAGGGGCAGGCAAACTAACTTAGGCCCGTCGTTGGGAATTCCTCGGATCACGGGGGCCGCGTGGCACTAGTCCTAACAAAAACTTTTTTGGAATTTGTAAAAGCGTCGAAAAATAAACCAATTGTTTACCAAT

**5. KR611565.*1 Fusarium oxysporum* strain NSF2**

CCTCNCCGCGTGTACTGGCTCGGCCGGGCCTTTCCCTCTGTGGAACCCCATGCCCTTCACTGGGCGTGGCGGGGAAACAGGACTTTTACTGTGAAAAAATTAGAGTGCTCCAGGCAGGCCTATGCTCGAATACATTAGCATGGAATAATAGAATAGGACGTGTGGTTCTATTTTGTTGGTTTCTAGGACCGCCGTAATGATTAATAGGGACAGTCGGGGGCATCAGTATTCAATTGTCAGAGGTGAAATTCTTGGATTTATTGAAGACTAACTACTGCGAAAGCATTTGCCAAGGATGTTTTCATTAATCAGGAACGAAAGTTAGGGGATCGAAGACGATCAGATACCGTCGTAGTCTTAACCATAAACTATGCCGACTAGGGATCGGACGGTGTTATTTTTTGACCCGTTCGGCACCTTACGAGAAATCAAAGTGCTTGGGCTCCAGGGGGAGTATGGTCGCAAGGCTGAAACTTAAAGAAATTGACGGAAGGGCACCACCAGGGGTGGAGCCTGCGGCTTAATTTGACTCAACACGGGGAAACTCACCAGGTCCAGACACAATGAGGATTGACAGATTGAGAGCTCTTTCTTGATTTTGTGGGTGGTGGTGCATGGCCGTTCTTAGTTGGTGGAGTGATTTGTCTGCTTAATTGCGATAACGAACGAGACCTTAACCTGCTAAATAGCCCGTATTGCTTTGTCAGTACGCTGGCTTCTTATAGGGACTATCGGCTCCGCCCGATGGAAGTTTGATGCAATAACAGGTCTGTGATGTCCTAATATGTTCTGGTCCGNACNCGCGCTACCCTGACCGAGGCAGCGAGTACTTCCTTGTCCGAGAAGGATGGGTAAGGTTGATT

**6. MF426031.1 *Fusarium proliferatum* strain AF04**

ACCCCTGTGACTACCAATTGTTGCCTCGGCGGATCAGCCCGCTCCCGGTAAAACGGGACGGCCCGCCAGAGGACCCCTAAACTCTGTTTCTATATGTAACTTCTGAGTAAAACCATAAATAAATCAAAACTTTCAACAACGGATCTCTTGGTTCTGGCATCGATGAATAACGCAGCAAAATGCGATAAGTAATGTGAATTGCAGAATTCAGTGAATCATCGAATCTTTGAACGCACATTGCGCCCGCCAGTATTCTGGCGGGCATGCCTGTTCGAGCGTCATTTCACCCCTCAAGCCCCCGGGTTTGGTGTTGGGGATCGGNGAGCCCTTGGGGCAAACCGGCGCCGCCCGAATTGCGGGGGCTCGTTGAT

**7. KP686186.1 *Trichoderma spirale* strain** **YIMPH30310**

TGTGACGTTACCAAACTGTTGCCTCGGCGGGATCTCTGCCCCGGGTGCGTCGCAGCCCCGGACCAAAGCGCCCGCCTGAGGACCAACCAAAACTCTTTTGTATACCCCCTCGCGGGTTTTTATATCTGAGCCATCTCGGCGCCTCTCGTAGGCGTTTCGAAAATGAATCAAAACTTTCATCAGCGGATCTCTTGGTTCTGGCAACGATGACGAACCCTCCTAAATGTGATAAGTAAGGGGATTTGCAGAATTCAGTGATTCATCTTATCTTTGAACTCTCATTGCGCCCGCGACCAGCCTGGCGGGACTGTGTGTCAGAGCGTCATTTCGCCCCTCAACCCCGGAGGGGGGTC

**8. EU409807.1 *Aspergillus nidulans* strain FH5**

TGCCTCCGGGCGCCGGGCTCCCACCCGTGACTACCTAACACTGTTGCTTCGGCGGGGAGCCCCCCAGGGGCGAGCCGCCGGGGACCACTGAACTTCATGCCTGAGAGTGATGCAGTCTGAGCCTGAATACAAATCAGTCAAAACTTTCAACAATGGATCTCTTGGTTCCGGCATCGATGAAGAACGCAGCGAACTGCGATAAGTAATGTGAATTGCAGAATTCAGTGAATCATCGAGTCTTTGAACGCACATTGCGCCCCCTGGCATTCCGGGGGGCATGCCTGTCCGAGCGTCATTGCTGCCCTCAAGCCCGGCTTGTGTGTTGGGTCGTCGTCCCCCCCGGGGGACGGGCCCGAAAGGCAGCGGCGGCACCGTGTCCGGTCCTCGAGCGTATGGGGCTTTGTCACCCGCTCGATTAGGGCCGGCCGGGCGCCAGCCGGCGTCTCCAACCTTATTTTTCTCAGGTTGACCTCGGATCAGGTAGGGATACCCGCTGAACTTAAGCATATCA

**9. KF358720.1 *Penicillium ochrochloron* strain PFR8**

CTGGGTCGGCCTCCCACCCGTGTTTATCGTACCTTGTTGCTTCGGCGGGCCCGCCTCACGGCCGCCGGGGGGCATCCGCCCCCGGGCCCGCGCCCGCCGAAGACACCATTGAACTCTGTCTGAAGATTGCAGTCTGAGCGATTAGCTAAATCAGTTAAAACTTTCAACAACGGATCTCTTGGTTCCGGCATCGATGAAGAACGCAGCGAAATGCGATACGTAATGTGAATTGCAGAATTCAGTGAATCATCGAGTCTTTGAACGCACATTGCGCCCCCTGGTATTCCGGGGGGCATGCCTGTCCGAGCGTCATTGCTGCCCTCAAGCACGGCTTGTGTGTTGGGCCCCGCCCCCCGGTTCCGGGGGGCGGGCCCGAAAGGCAGCGGCGGCACCGCGTCCGGTCCTCGAGCGTATGGGGCTTTGTCACCCGCTCTGTAGGCCCGGCCGGCGCCCGCCGGCNACCCCAAATCAATCTATCCAGGTTGACCTCGGATCAGGTAGGGATACCCGCTGAACTTAAGCATATG

# 10. AB105434.1 *Pichia guilliermondii* strain IAM 14500

CTTTCCTTCTGGCTAACCATTCGCCCTTGTGGTGTTTGGCGAACCAGGACTTTTACTTTGAAAAAATTAGAGTGTTCAAAGCAGGCCTTTGCTCGAATATATTAACATGGAATAATAAAATAGGACGTTATGGTTCTATTTTGTTGGTTTCTAGGACCATCGTAATGATTAATAGGGACGGTCGGGGGCATCAGTATTCAGTTGTCAGAGGTGAAATTCTTAGATTTACTGAAGACTAACTACTGCGAAAGCATTTGCCAAGGACGTTTTCATTAATCAAGAACGAAAGTTAGGGGATCGAANGTGATCAAATACCGTCGTAGTCTTAACCATAAACTATGCCGACTAGGGATCGGGTGTTGTTCTTTTTTTGACGCACTCNGCACCTTACGAGAAATCAAAGTCTTTGGGTTCTGGGGGGAGTATGGTCGCAAGGCTGAAACTTAAAGGAATTGACGGAAGGGCACCACCAGGAGTGGAGCCTGCGGCTTAATTTGACTCAACACGGGGAAACTCACCAGGTCCAGACACAATAAGGATTGACAGATTGAGAGCTCTTTCTTGATTTTGTGGGTGGTGGTGCATGGCCGTTCTTAGTTGGTGGAGTGATTTGTCTGCTTAATTGCCATAACTAAACGAAGATCTTTAACCTACTAAATAGTGCTGCCAAGCTTTTGCTGGGCATAGGCACTTTCTAAAGGGGACTATCGAATTTCAAACGGATGGGAAATTTGAGGCATTAAC

# *11*. KX650827.1 *Nigrospora sp. isolate DL2*

CNAACCCATGTGACATATCTCTTTGTTGCCTCGGCGCAAGCTACCCGGGACCTCGCGCCCCGGGCGGCCCGCCGGCGGACAAACCAAACTCTGTTATCTTCGTTGATTATCTGAGTGTCTTATTTAATAAGTCAAAACTTTCAACAACGGATCTCTTGGTTCTGGCATCGATGAAGAACGCAGCGAAATGCGATAAGTAATGTGAATTGCAGAATTCAGTGAATCATCGAATCTTTGAACGCACATTGCGCCCATTAGTATTCTAGTGGGCATGCCTGTTCGAGCGTCATTTCAACCCCTAAGCACAGCTTATTGTTGGGCGTCTACGTCTGTAGTGCCTCAAAGACATTGGCGGAGCGGCAGCAGTCCTCTGAGCGTAGTAATTCTTTATCTCGCTTCTGTTAGGCGCTGCCCCCCCGGCCGTAAAACCCCCAATTTTTTCTGGTTGACCTCGGATCAGGTAGGAATACCCGCTGAACTTAAGCATATCAATAA

**12. KF360838.1 *Periconia macrospinosa* strain KS00113**

CGCCGCTCCTTATACACCCACCCTCTGCCTACGTGTACCTCTATAGCTTCCTCGGCGGGCTCGCCCGCCGCCAGGAACCCACGAAACCCCTTGCATTATACGCGAAAACTTCTGATAACAAACCTAAATTATCACAACTTTCAACAATGGATCTCTTGGTTCTGGCATCGATGAAGAACGCAGCGAAATGCGATAAGTAGTGTGAATTGCAGAATTCAGTGAATCATCGAATCTTTGAACGCACATTGCGGCCATAGGTATTCCTTTGGCCATGCCTGTTCGAGCGTCATTTACACCCTCAAGCCTAGCTTGGTGTTGGGCGTCTGTCCCGCCGTTCTCGCGCGCGGACTCGCCTCAAAGTCATTGGCGGCGGTCGTGCCGGCCCCCTCGCGCAGCACATTTGCGCTTCTCGGAGGCCCGGCGGATCCGCGCTCCAGCAAGACCTTTCACGACTTGACCTCGGATCAGGTAGGGATACCCGCTGAACTTAAGCATATCAATAAGCGCCGGAAGGGTTTGTAGGCGTTGAGGGGGGTTTCGGGG

**13. KX066060.1 *Aspergillus fumigatus* strain FZ 18**

GCCCCTGGGTCACCTCCCACCCGTGTCTATCGTACCTTGTTGCTTCGGCGGGCCCGCCGTTTCGACGGCCGCCGGGGAGGCCCTGCGCCCCCGGGCCCGCGCCCGCCGAAGACCCCAACATGAACGCTGTTCTGAAAGTATGCAGTCTGAGTTGATTATCGTAATCANTTAAAACTTTCAACAACGGATCTCTTGGTTCCGGCATCGATGAAGAACGCAGCGAAATGCGATAAGTAATGTGAATTGCAGAATTCAGTGAATCATCGAGTCTTTGAACGCACATTGCGCCCCCTGGTATTCCGGGGGGCATGCCTGTCCGAGCGTCATTGCTGCCCTCAAGCACGGCTTGTGTGTTGGGCCCCCGTCCCCCTCTCCCGGGGGACGGGCCC

**14. KY225624.1 *Trichoderma atroviride* strain QT21978**

ACCAAACTGTTGCCTCGGANGGGGTCACGCCCCGGGTGCGTCGCAGCCCCGGAACCAGGCGCCCGCCGGAGGGACCAACCAAACTCTTTTCTGTAGTCCCCTCGCGGACGTTATTTCTTACAGCTCTGAGCAAAAATTCAAAATGAATCAAAACTTTCAACAACGGATCTCTTGGTTCTGGCATCGATGAAGAACGCAGCGAAATGCGATAAGTAATGTGAATTGCAGAATTCAGTGAATCATCGAATCTTTGAACGCACATTGCGCCCGCCAGTATTCTGGCGGGCATGCCTGTCCGAGCGTCATTTCAACCCTCGAACCCCTCCGGGGGGTCGGCGTTGGGGACCTCGGGAGCCCCTAAGACGGGATCCCGGCCCCGAAATACAGTGGCGGTCTCGCCGCAGCCTCTCCTGCGCAGTAGTTTGCACAACTCGCACCGGGAGCGCGGCGCGTCCACGTCCGTAAAACACCCAACTTCTGAAATGTTGACCTCGGATCAGGTAGGAATACCCGCTGAACTTAAGCATATCAATAAGCGGCCGAA

**15. GQ924020.1 *Pleosporales* clone K1Fc722H**

TGCAAGCCTTCGTCTACACCCATGTCTTTTGCGTACTTCTTGTTTCCTCGGTGGCGCAAGCCGCCGATTGGACAAACCAAAACCTTTTTTGTAATTGCAATCAGCGTCTGAAAATAATCTAATTATTTACAACTTTCAACAACGGATCTCTTGGTTCTGGCATCGATGAAGAACGCAGCGAAATGCGATAAGTAGTGTGAATTGCAGAATTCAGTGAATCATCGAATCTTTGAACGCACATTGCGCCCCTTGGTATTCCATGGGGCATGCCTGTTCGAGCGTCATTTGTACCCTCAAGCTTTGCTTGGTGTTGGGCGTCTTGTCGTATTACGACTCGCCTTAAATACATTGGCAGCCGGCACTTTGGCCTAGGAGCGCAACACATTTTGCGATCGTAGCCCGTTGTACTGGCGTCCATCAAGAACATTTACCACGTTTGACCTCGGATCAGGTAGGGATACCCGCTGAACTTAAGCATATCAATAAGCGGAGGCCTATTGTTATCAAA

**16. FJ914711.1 *Rhinocladiella* sp. HSAUP074099**

ATTGTTTATGATACGAACGTGTTGCTTCGGTAGGCCTGGTCTCTACCTGCTGGGGGGCCGTCACACGCCCGCCGGAGAGTGCCTGCCGACAGCCTAAACCTCAAAATCTTTAACCAAACGTGTCTTTGTCTGAGTAAACGTCTTTAATAAAAGCAAAACTTTCAACAACGGATCTCTTGGTTCTGGCATCGATGAAGAACGCAGCGAAATGCGATAAGTAATGCGAATTGCAGAATTCTCGTGAGTCATCGAATCTTTGAACGCACATTGCGCCCTTTGGTATTCCGAAGGGCATGCCTGTTCGAGCGTCATTTTCACCCCTCAAGCCCCCGGCTTGGTGTTGGACGGTTTGGTCCAGGGCCCCCCCTGGACCCCTCCCAAAGACAATGACGGCGGGCTGTTGCACCCCCGGTACACTGAGCATCTTCACGGAGCACGTACCGGTCTCAAGGGTCGACGGCACCCGGTCTACACCTATATCTTTCACAAGGTTGACCTCGGATCAGGTAGGAATACCCGCTGAACTTAAGCATATCAATAAGCGGAGCA

**17. AB176623.1 *Talaromyces helices* strain CBS 652.66**

CCGNGTCNATAATTACACCTGTTGCTTTGAGCGGGCCCACCGGGGCCACCTGGTCGCCGGGGGACGCCTGTCCCCGGGCCCGCGCCCGCCGAAGCACCCCCTGAACTCTGAAGAAGATCGGGCTGTCTGAGTACCTAAAAAATTGTCAAAACTTTCAACAATGGATCTCTTGGTTCCGGCATCGATGAANAACGCAGCAAAATGCGATAAGTAATGTGAATTGCAAAATTCCGTGAATCATCGAATCTTTGAACGCACATTGCGCCCCCTGGAATTCCGGGGGGCATGCCTGTCCGAGCGTCATTTCTGCCCTCAAGCACGGCTTGTGTGTTGGGTGTGGTCCCCCCGGGGACCTGCCCCANAGGCAGCGGCGACGCCCGTCGGGTCCTCAAGCGCATGGGGCTTTGTCACTCGCTCGGGAGGGACCTGCGGGGGTTGGTCACCACTCTTTCTATTTTTTTTAACGTTGACCTCGGATCATG

**18. [KU561870.1](https://www.ncbi.nlm.nih.gov/nucleotide/KU561870.1?report=genbank&log$=nucltop&blast_rank=1&RID=SNA7K3P501R) *Pleosporales sp. isolate EDSHB10***

GGACCGTCGCTCGCGACGACGCTGCCTTGGGCGCTTAGCCCTTGACTATCACCTTGACTACGTGCACCTTTTGTTGTTTCCTCCGCAGGTCCTCTGCCGCCAGGAACCCCCCAAACCCTTTTGCTACAGCATCCGAACTTCTGAAGGCAACCAGATCATTTACAACTTTGAACATTGGATCTCTTGGTTCTGGTATCTATGAGGAAAGCGGCCAAATGCGATAAGTATTGAGAATTGAACAATTCGGGAATCATCGAATCTTTGAAGGCACATTGCCCCCAATGGTATTCCATGGGGCATGCCTGTTCGAGCGCCATTGACCCCCTCATGCCCCACTTGGTGTTGGTGATCTGGATAGGTTAGCGATCGTATTCGGTCAAATTGTGTAACAACGGGTTGTCCAGTTTCAGCTCAAAAATGACGGTCTTCAGAGTAGAGCCCCGGCTACCTACCGGTCACAATCCGC

**19. JQ761839.1 *Hypoxylon sp.* genotype 520 isolate NC1198**

TTGTGACCTTACTGTCGTTGCCTCGGCGTGAGCTACGGCTACCCTGTAGCTACCCTATAGCTACCCTGCAGCTACCCTATAGTTGACCAGTAGCTACCCTGTAGTTACCCTATAGTTACCCTGCAGCTACCCTATAGTTGACCAGTAGCTACCCTGTAGCCGGCTTATGGCCCGCCGAAGGACCGCTAAACTCTTGTTTTTTACCACTGTTTCTCTGAATTTTAAACTTAAATAAGTTAAAACTTTCAACAACGGATCTCTTGGTTCTGGCATCGATGAAGAACGCAGCGAAATGCGATAAGTAATGTGAATTGCAGAATTCAGTGAATCATCGAATCTTTGAACGCACATTGCGCCCATTAGTATTCTAGTGGGCATGCCTATTCGAGCGTCATTTCGACCCTTAAGCCCCTGTTGCTTAGCGTTGGGAATCTACAGCGTAGTTCCTTAAAGTTAGTGGCGGAGTTAGGGTACACTCTCAGCGTAGTAATCTTTCTCGCTCGTGTGGTGGCCCTGGCTGCTAGCCGTTAAACCCCTATATTTTCTAGTGGTTGACCTCGGATTAGGTAGGAATACCCGCTGAACTTAAGCATATCAATAAGCGGAG

**20. GQ923961.1 *Pleosporales* sp. G9i87H**

GCTCGGGGGACCCGGTCGCTCGCGACGACGCTGCCTTGGGCGCTTAGCCCTTGACTATCACCTTGACTACGTGCACCTTTTGTTGTTTCCTCGGCAGGTCCTCTGCCGCCAGGAACCCCCCAAACCCTTTTGCAACAGCATCCAAACTTCTGAAAACAAACCAAATCATTTACAACTTTTAACAATGGATCTCTTGGTTCTGGCATCGATGAAGAACGCAGCGAAATGCGATAAGTAGTGTGAATTGCAGAATTCAGTGAATCATCGAATCTTTGAACGCACATTGCGCCCCATGGTATTCCGTGGGGCATGCCTGTTCGAGCGTCATTTACCCCCTCAAGCTCCGCTTGGTGTTGGGCGTCTGTCCCGCTTCGCGCGCGGACTCGCCCCAAAGGTATTGGCAGCGGTCGTGCCAGCTTCTCGCGCAGCACATTGCGCTTCTCGAGGCACCGGTGGGCCCGCGTCCATCAAGCTCACCCCCCCAGTTTGACCTCGGATCAGGTAGGGATACCCGCTGAACTTAAGCATATCAATAAGCGGAGGAA

**21. EU784644.*1 Pichia guilliermondii* strain CXF-1**

TACTGNGACCCNACCGAGCCTTTCCTTCTGGCTAACCATTCGCCCTTGTGGTGTTTGGCGAACCAGGACTTTTACTTTGAAAAAATTAGAGTGTTCAAAGCAGGCCTTTGCTCGAATATATTAGCATGGAATAATAGAATAGGACGTTATGGTTCTATTTTGTTGGTTTCTAGGACCATCGTAATGATTAATAGGGACGGTCGGGGGCATCAGTATTCAGTTGTCAGAGGTGAAATTCTTAGATTTACTGAAGACTAACTACTGCGAAAGCATTTGCCAAGGACGTTTTCATTAATCAAGAACGAAAGTTAGGGGATCGAAGATGATCAGATACCGTCGTAGTCTTAACCATAAACTATGCCGACTAGGGATCGGGTGTTGTTCTTTTTTTGACGCACTCGGCACCTTACGAGAAATCAAAGTCTTTGGGTTCTGGGGGGAGTATGGTCGCAAGGCTGAAACTTAAAGGAATTGACGGAAGGGCACCACCAGGAGTGGAGCCTGCGGCTTAATTTGACTCAACACGGGGAAACTCACCAGGTCCAGACACAATAAGGATTGACAGATTGAGAGCTCTTTCTTGATTTTGTGGGTGGTGGTGCATGGCCGTTCTTAGTTGGTGGAGTGATTTGTCTGCTTAATTGCGATAACGAACGAGACCTTAACCTACTAAATAGTGCTGCTAGCTTTTGCTGGTATAGTCACTTTCTTANAGGGACTATCGATTTCAAGTCGATGGAAGTTTGAGGNAATAACATTTCTGTGATGCCCTTAGACGTTCTGGGGCCGCACGCGCGCTACACTGACGGAGCCAGCGAGTATNACCTTGGGCCGATAGGTCTGNGAAATTTTGTGAACTCCGTCGTGCTGGGGATANAGCATTCGNATTATTGCTCTTCAACCAAGAATTTCTAGGAGNCNCCAGGNCATCACCGTTGCGTTGATTACCGTGCCGGCCCTTTGTACACCACCGCCCGTCCCTACTTACCAAATTGGATGGNTCAATGAGGCCTCTTTGGATCGGTT

# 22. MF061769.1 *Fusarium chlamydosporum* strain SCAU107

TCCGAACCCCTGTGACATACCTATACGTTGCCTCGGCGGATCAGCCCGCGCCCCGTAAAACGGGACGGCCCGCCCGAGGACCCCTAAACTCTGTTTTTAGTGGAACTTCTGAGTAAAACAAACAAATAAATCAAAACTTTCAACAACGGATCTCTTGGTTCTGGCATCGATGAAGAACGCAGCAAAATGCGATAAGTAATGTGAATTGCAGAATTCAGTGAATCATCGAATCTTTGAACGCACATTGCGCCCGCCAGTATTCTGGCGGGCATGCCTGTTCGAGCGTCATTTCAACCCTCAAGCTCAGCTTGGTGTTGGGACTCGCGGTAACCCGCGTTCCCCAAATCGATTGGCGGTCACGTCGAGCTTCCATAGCGTAGTAATCATACACCTCGTTACTGGTAATCGTCGCGGCCACGCCGTTAAACCCCAACTTCTGAATGTTGACCTCGGATCAGGTAGGAATACCCGCTGAACTTAAGCATATCAATAAGCGGAGGAAAACCCCGGAGAAAAG

**23. HM037942.1 *Pichia guilliermondii* strain wxm69**

TGCCTCTATAAATATTGGAAAATAGCTGTGTGTGTTTATATATATATTTAGGAAGGGGAGGGACGTGTGAGTGTGTGGGCCTAAGGTTTAACAAAATATAGATTTGCGAGAGTACATGTTTANGGATTTTGGGAAACGAAGGAAACTTTAAACAACGGATCTCTTGGTTCTCCCATCNATGAACAACGCAGCGAAATGCGATAAGTAATATGAATTGCANATTTTCGTGAATCATCGAATCTTTGAACGCACATTGCGCCCTCTGGTATTCCNGAGGGCATGCCTGTTTGAGCGTCATTTCTCTCTCAAACCCCCGGGTTTGGTATTGAGTGATACTCTTAATCGGACTAGGCGTTTGCTTGAAAAGTATTGGCATGGGTAGTACTANATAGTGCTGTCGACCTCTCAATGTATTAGGTTTATCCAACTCGTTGAATGGTGTGGCGGGATATTTCTGGTATTGTTGGCCCGGCCTTACAACAACCAAACTAGTTTGACCTCNAATCAGGTAGGAATACCCGCTGAACTTAACCATATCAATAANCGGAGGAACGG

**24. FJ840490.1 *Aspergillus fumigatus* strain FS160**

GGNCGCCTCCCCGCGAGTACTGGTCCGGCTGGACCTTTCCTTCTGGGGAACCTCATGGCCTTCACTGGCTGTGGGGGGAACCAGGACTTTTACTGTGAAAAAATTAGAGTGTTCAAAGCAGGCCTTTGCTCGAATACATTAGCATGGAATAATAGAATAGGACGTGCGGTTCTATTTTGTTGGTTTCTAGGACCGCCGTAATGATTAATAGGGATAGTCGGGGGCGTCAGTATTCAGCTGTCAGAGGTGAAATTCTTGGATTTGCTGAAGACTAACTACTGCGAAAGCATTCGCCAAGGATGTTTTCATTAATCAGGGAACGAAAGTTAGGGGATCGAAGACGATCAGATACCGTCGTAGTCTTAACCATAAACTATGCCGACTAGGGATCGGGCGGTGTTTCTATGATGACCCGCTCGGCACCTTACGAGAAATCAAAGTTTTTGGGTTCTGGGGGGAGTATGGTCGCAAGGCTGAAACTTAAAGAAATTGACGGAAGGGCACCACAAGGCGTGGAGCCTGCGGCTTAATTTGACTCAACACGGGGAAACTCACCAGGTCCAGACAAAATAAGGATTGACAGATTGAGAGCTCTTTCTTGATCTTTTGGATGGTGGTGCATGGCCGTTCTTAGTTGGTGGAGTGATTTGTCTGCTTAATTGCGATAACGAACGAGACCTCGGCCCTTAAATAGCCCGGTCCGCATTTGCGGGCCGCTGGCTTCTTANGGGGACTATCGGCTCAAGCCGATGGAAGTGCGCGGCAATAACAGGTCTGTGATGCCCTTAGATGTTCTGGGCCGCACGCGCGCTACACTGACAGGGNCAGCGAGTACATCACCTTGGCCGAGAGGTCTGGGTAATCTTGTTAAACCCTGTCGTGCTGGGGATAGAGCATTGCAATTATTGCTCTTCAACGAGGAATGCCTACTAGGCACGAGTCATCAGCTCGNGCCGATTACGTCCCTGCCCTTTGNACACACCGCCCGTCGCTACTACCGATTGAATGGCTCG

**25. KU702683.1 *Penicillium simplicissimum* isolate BK227**

CTGGGTCACCTCCCACCCGTGTTTATCGTACCTTGTTGCTTCGGCGGGCCCGC

CTCACGGCCGCCGGGGGGCATCCGCCCCCGGGCCCGCGCCCGCCGAAGACACCATTGAACTCTGTCTGAAGATTGCAGTCTGAGCGATTAGCTAAATCAGTTAAAACTTTCAACAACGGATCTCTTGGTTCCGGCATCGATGAAGAACGCAGCGAAATGCGATACGTAATGTGAATTGCAGAATTCAGTGAATCATCGAGTCTTTGAACGCACATTGCGCCCCCTGGTATTCCGGGGGGCATGCCTGTCCGAGCGTCATTGCTGCCCTCAAGCACGGCTTGTGTGTTGGGCCCCGCCCCCCGGTTCCGGGGGGCGGGCCCGAAAGGCAGCGGCGGCACCGCGTCCGGTCCTCGAGCGTATGGGGCTTTGTCACCCGCTCTGTAGGCCCGGCCGGCGCCCGCCGGCGACCCCAAATCAATCTATCCAGGTTGACCTCGGATCAGGTAGGGATACCCGCTGAACTTA

**26. GU073119.1 *Leptosphaerulina chartarum* strain DH08111**

GCGGCGCGGCCCCCGAGGAGCGGANCAATCCTTGGGAGGTATGCGGGGGCTTCGAGCCCCCCATTTACGCACGCACGACTGCCATCCTTACTTTACGAGCACCTTCTGTTCTCCCTCGGCGGGGCAACCTGCCGTTGGAACCGAATAAACTCTTTTTGCATCTAGCATTACCTGTTCCGAAACAAACAATCGTTACAACTTTCAACAATGGATCTCTTGGCTCTGGCATCGATGAAGAACGCAGCGAAATGCGATAAGTAGTGTGAATTGCAGAATTCAGTGAATCATCGAATCTTTGAACGCACATTGCGCCCCTCGGTATTCCGTGGGGCATGCCTGTTCGAGCGTCATCTACACCCTCAAGCTCTGCTTGGTGTTGGGCGTCTGTCCCGCCTCCGCGCGTGGACTCGCCCCAAATTCATTGGCAGCGGTCCTTGCCTCCTCTCGCGCAGCACATTGCGCTTCTCGAGGGGCTACGGCTCGCGTCCAACAAGCACATTTACCGTCTTTGACCTCGGATCAGGTAGGGATACCCGCTGAACTTAAGCATATCAATAAGCNGAAGAAANNNTCATATTC

**27. AB473810.1 *Fusarium solani strain THIF01***

CGCCTCACCGCGTGTACTGGTCCGGCCGGGCCTTTCCCTCTGTGGAACCCCATGCCCTTCACTGGGTGTGGCGGGGAAACAGGACTTTTACTGTGAAAAAATTAGAGTGCTCCAGGCAGGCCTATGCTCGAATACATTAGCATGGAATAATAGAATAGGACGTGTGGTTCTATTTTGTTGGTTTCTAGGACCGCCGTAATGATTAATAGGGACAGTCGGGGGCATCAGTATTCAATTGTCAGAGGTGAAATTCTTGGATTTATTGAAGACTAACTACTGCGAAAGCATTTGCCAAGGATGTTTTCATTAATCAGGAACGAAAGTTAGGGGATCGAAGACGATCAGATACCGTCGTAGTCTTAACCATAAACTATGCCGACTAGGGATCGGACGGTGTTATATTTTGACCCGTTCGGCACCTTACGAGAAATCAAAGTGCTTGGGCTCCAGGGGGAGTATGGTCGCAAGGCTGAAACTTAAAGAAATTGACGGAAGGGCACCACCAGGGGTGGAGCCTGCGGCTTAATTTGACTCAACACGGGGAAACTCACCAGGTCCAGACACAATGAGGATTGACAGATTGAGAGCTCTTTCTTGATTTTGTGGGTGGTGGTGCATGGCCGTTCTTAGTTGGTGGAGTGATTTGTCTGCTTAATTGCGATAACGAACGAGACCTTAACCTGCTAAATAGCCCGTATTGCTTTGGCAGTACGCTGGCTTCTTAGAGGGACTATCGGCTCAAGCCGATGGAAGTTTGAGGCAATAACAGGTCTGTGATGCCCTTAGATGTTCTGGGCCGCACGCGCGCTACACTGACGGAGCCAGCGAGTACTTCCTTGGCCGAAAGGCCCGGGTAATCTTGTTAAACTCCGTCGTGCTGGGGATAGAGCATTGCAATTATTGCTCTTCCACGAGGAATCCCTAGTAAGCGCAAGTCATCAGCTTGCGTTGATTACGTCCCTGCCCTTTGTACACACNGCCCGTCGCTACTACCGATTGAATGGCTCANTGATGCGTCCGNACTGGCCCCCGCCGGTGGGCATTACCGCTCTGGCGGGAAACTCTACC

**28. GU138648.1 *Chaetomium globosum* strain PF-1**

ACTCCCGGACCGTTGTGACGTTACCTATACCGTTGCTTCGGCGGGCGGCCCCGGGGTTTACCCCCCGGGCGCCCCTGGGCCCCACCGCGGGCGCCCGCCGGAGGTCACCAAACTCTTGATAATTTATGGCCTCTCTGAGTCTTCTGTACTGAATAAGTCAAAACTTTCAACAACGGATCTCTTGGTTCTGGCATCGATGAAGAACGCAGCGAAATGCGATAAGTAATGTGAATTGCAGAATTCAGTGAATCATCGAATCTTTGAACGCACATTGCGCCCGCCAGCATTCTGGCGGGCATGCCTGTTCGAGCGTCATTTCAACCATCAAGCCCCCGGGCTTGTGTTGGGGACCTGCGGCTGCCGCAGGCCCTGAAAAGCAGTGGCGGGCTCGCTGTCGCACCGAGCGTAGTAGCATACATCTCGCTCTGGTCGCGCCGCGGGTTCCGGCCGTTAAACCACCTTTTAACCCAAGGTTGACCTCGGATCAGGTAGGAAGACCCGCTGAACTTAAGCATATCAATACCGGATCGGAA
